# The Landscape of Lipid Metabolism in Lung Cancer: The Role of Structural Profiling

**DOI:** 10.3390/jcm12051736

**Published:** 2023-02-21

**Authors:** Chanchan Hu, Luyang Chen, Yi Fan, Zhifeng Lin, Xuwei Tang, Yuan Xu, Yiming Zeng, Zhijian Hu

**Affiliations:** 1 Department of Epidemiology and Health Statistics, School of Public Health, Fujian Medical University, Fuzhou 350122, China; 2 Key Laboratory of Ministry of Education for Gastrointestinal Cancer, Fujian Medical University, Fuzhou 350122, China; 3 Department of Respiratory Pulmonary and Critical Care Medicine, The Second Affiliated Hospital of Fujian Medical University, Quanzhou 362000, China; 4 Respiratory Medicine Center of Fujian Province, Quanzhou 362000, China; 5 Clinical Research Center, The Second Affiliated Hospital of Fujian Medical University, Quanzhou 362000, China

**Keywords:** lung cancer, lipid structure profiling, inflammation, metabolic, biomarkers, n-3 PUFA

## Abstract

The aim of this study was to explore the relationship between lipids with different structural features and lung cancer (LC) risk and identify prospective biomarkers of LC. Univariate and multivariate analysis methods were used to screen for differential lipids, and two machine learning methods were used to define combined lipid biomarkers. A lipid score (LS) based on lipid biomarkers was calculated, and a mediation analysis was performed. A total of 605 lipid species spanning 20 individual lipid classes were identified in the plasma lipidome. Higher carbon atoms with dihydroceramide (DCER), phosphatidylethanolamine (PE), and phosphoinositols (PI) presented a significant negative correlation with LC. Point estimates revealed the inverse associated with LC for the n-3 PUFA score. Ten lipids were identified as markers with an area under the curve (AUC) value of 0.947 (95%, CI: 0.879–0.989). In this study, we summarized the potential relationship between lipid molecules with different structural features and LC risk, identified a panel of LC biomarkers, and demonstrated that the n-3 PUFA of the acyl chain of lipids was a protective factor for LC.

## 1. Introduction

Lung cancer (LC) is the second most common cancer and the leading cause of cancer-related death worldwide [1]. Despite several initiatives to control tobacco, there has been no significant downward trend in incidences of LC. There are no or obvious specific symptoms in the early stages of LC, and most people are diagnosed in the mid- to late- stages. This creates a substantial burden for healthcare systems and severely compromises the quality of life of people with LC.

LC has various pathogenic factors and complex pathological mechanisms, but metabolic reprogramming is one of the most important hallmarks of tumor cells. It is commonly found in the process of glucose metabolism, amino acid metabolism, and lipid metabolism, while changes in lipid metabolism have received less attention compared with other topics. Lipids are essential components of the biological membranes and structural units of cells. A comprehensive classification system organizes lipids into these eight well-defined categories: fatty acyls (FA), glycerolipids (GL), glycerophospholipids (GP), sphingolipids (SP), sterol lipids (ST), prenol lipids (PR), saccharolipids (SL), and polyketides (PK) [2]. Lipid metabolism is critical in the development of tumorigenesis [3,4]. Recently, research interests have shifted toward using omics to explore the pathophysiological processes involved in the development of LC, where lipidomics captures changes in endogenous and exogenous molecules that confer further insights [5]. The dysregulation of lipid metabolism is one of the most prominent metabolic alterations in LC which could lead to abnormal gene expression and disorders of signaling pathways [6]. Research on major lung cell types isolated from human donors illustrated the significant role of lipids in lung functions and lung development, including phosphoglycerol (PG), diacylglyceride (DAG), and triacylglyceride (TAG) [7]. In addition, research on lipidomics for LC [3,8,9] has become more common, but it merely provides clues regarding the importance of lipid classes, including phosphatidylcholine (PC), PE, and lysophosphatidylcholine (LPC), in the pathology of non-small cell lung cancer (NSCLC). FAs are at the root of these complex lipids, and their functions and features are mainly determined by their structure, which depends on the number of carbons in the chain (short, medium, long, or extra-long fatty acids) and the number of double bonds (saturated, monounsaturated, and polyunsaturated fatty acid (PUFA)) [10]. Changes in saturated and unsaturated fatty acid levels can disrupt homeostasis in vivo, enhance cellular stress, alter cell membrane dynamics, and affect the uptake and efficacy of chemotherapeutic drugs [11,12]. Due to the structural and biosynthetic complexity of lipids, the results of previous studies are varied [3,13], and the contribution of lipid structural features in the pathogenesis of LC remains unexplored.

The identification of biomarkers or novel metabolic dysregulation pathways has long been a popular field. Thousands of candidate cancer biomarkers have been identified, but only a few are currently used in clinical practice. Lipid metabolism disorders have been found to have great potential for discovering biomarkers and understanding the pathogenesis of LC.

Chronic inflammation is a precondition for the progression of cancers [14]. Although numerous studies [15,16] have reported that inflammation-driven markers, including the neutrophil-lymphocyte ratio (NLR) and the platelet lymphocyte ratio (PLR), play a key role in tumorigenesis and progression, it is unknown whether inflammatory mediators play a role in the pathogenesis of LC caused by lipid metabolism disorders.

Herein, we aim to descriptively summarize the potential relationship between lipid molecules with different structural features and LC risk, identify prospective biomarkers of LC that can be applied in clinical diagnosis and treatment, and further discuss how inflammation mediates the relationship between lipids and LC risk.

## 2. Materials and Methods

### 2.1. Study Population

As part of an ongoing hospital-based case control study, patients were recruited from the Second Affiliated Hospital of Fujian Medical University. The inclusion criteria for the cases were as follows: (1) patients with diagnosed primary LC by fiberoptic bronchoscopy or histopathologic evaluation; (2) patients who have not received chemotherapy; and (3) patients without other lung diseases or systemic diseases, such as heart, liver, kidney, cranial, or brain. The exclusion criteria included patients with a pathologic diagnosis of lung inflammation, benign lesion, or secondary LC. During the same study period, 62 age-matched (±2 years) and sex-matched cancer-free healthy controls (median age: 54 years) were randomly selected from a health examination cohort. All subjects were Han Chinese people who had lived in Fujian for at least 10 years, did not suffer from coronary atherosclerotic heart disease, cerebrovascular disease, thyroid insufficiency, diabetes, or hyperlipidemia, and were able to answer the study questions. All participants provided written and informed consent. The present study was approved by the Second Affiliated Hospital of Fujian Medical University’s Institutional Review Board with the certificate number IRB No. 2021–452.

### 2.2. Chemicals and Reagents

The internal standards were purchased from AB SCIEX (refer to Supplementary Materials File S1 for details). HPLC grade methanol, isopropanol (IPA), acetonitrile (ACN), and water were purchased from Merck (Darmstadt, Germany).

### 2.3. Sample Collection and Preparation

Approximately 10 mL of peripheral venous blood was collected from each study subject under fasting conditions, fractioned by a trained researcher according to standard protocols, and the EDTA plasma was stored at −80 °C in deep freezers. Plasma samples were processed according to the following sequential steps: (1) 225 μL methanol was added to each of the 20 μL plasma samples and then vortexed for 10 s; (2) 13 μL internal standards and 750 μL MTBE were added, vortexed for 10 s, and then stood for 30 min; (3) an additional 188 μL water was added, vortexed for 20 s and then stood for 10 min before centrifugation at 15,000 rpm at 4 °C for 15 min; (4) 700 μL supernatant was collected from each sample separately and blow dried with a nitrogen blower; (5) 100 μL reconstitution reagent (65% ACN, 30% IPA, and 5% H2O, *v:v:v*) was added to each dried sample, then vortexed for 10 s, and centrifuged at 14,000 rpm at 4 °C for 10 min; and (6) 100 μL supernatant from each tube was then transferred into vials for HPLC-MS/MS analysis. Before sequence analysis, four consecutive quality control (QC) samples were injected to assess the reproducibility of the system. QC samples were inserted every 10 samples to evaluate the stability of the system in the sequence analysis.

### 2.4. HPLC-MS/MS Analysis

Seminal plasma lipidomics were detected using a HPLC coupled with 4500 QTRAP mass spectrometry (AB SCIEX Pte. Ltd., Framingham, MA, USA). Chromatographic separations were performed on an ACQUITY UPLC BEH HILIC column (1.7 μm, 100 mm × 2.1 mm, 186003461) (Waters). Its parameters are listed in Table S1.

Quantitation was performed on a 4500 QTRAP tandem mass spectrometer coupled with an electrospray ionization (ESI) source (the conditions of the ESI are shown in Table S2). Multiple reaction monitoring (MRM) was used to quantify the compounds in the positive and negative ion modes (Table S3 sheet 1–2 present detailed information about lipids in positive and negative modes). The ion adduct form of each lipid is shown in Table S3 sheet 3.

Throughout the analysis process, the samples were placed in an autosampler and analyzed continuously in a randomized order to avoid fluctuation in the instrument’s detection signal that would affect the experimental results.

### 2.5. Data Processing and Statistical Analysis

Peak identification, peak filtering, peak alignment, and lipid identification were performed using SCIEX OS software to obtain a two-dimensional data matrix, including the mass-to-nucleus ratio, retention time, peak area, and lipid class information. The relative abundance of each lipid was measured while considering the total area of all the transformations that were analyzed. The peak area data from the individual lipids were then transformed and normalized.

Since the relationship between lipids and disease risk may vary depending on the length and unsaturation of the acyl chain [17], lipid subclasses were grouped and further analyzed based on the carbon atom and double bond number. Twenty lipid subclasses were grouped by their total carbon number and total double bond number. The total concentrations of each group in the individual lipid subclasses were log-transformed and standardized into unit variance. Odds ratios (ORs) of LC risk per log-transformed SD increase in each lipid species, based on the number of carbon atoms and double bonds, were calculated using conditional logistic regression models and visualized by bubble plots. To further reveal and clarify the potential biological mechanisms of lipid species based on FA, the n-3 and n-6 PUFA scores among the subclass of lipid profiling were calculated, and the relationships were explored. Univariate and multivariate statistical analyses were used to screen the differential lipids. Univariate statistical analyses included the *t*-test, fold change (FC) analysis, and volcano plots based on the first two analyses. Multivariate statistical analysis included unsupervised principal component analysis (PCA), supervised partial least squares-discriminant analysis (PLS-DA), orthogonal partial least squares-discriminant analysis (OPLS-DA), and sparse partial least squares-discriminant analysis (sPLS-DA). Then, two machine learning approaches (including the random forest algorithm and support vector machine algorithm) were used to define a combinational lipid biomarker in the plasma samples to distinguish patients with LC from healthy controls. Lipid scores (LS) were calculated by multiplying the OR of the ten biomarkers by their corresponding concentration values. LS = 0.23 × DAG (32:0) + 0.28 × DAG (34:0) + 0.27 × FFA (16:2) + 0.20 × FFA (24:1) + 0.17 × PE (O-38:5) + 0.32 × PC (40:4) + 0.16 × PS (38:6) + 0.30 × TAG (55:2/FA 18:2) + 0.26 × TAG (54:7/FA 18:1) + 0.25 × DAG (40:8). A mediation analysis [18] was performed to test whether the observed associations between LS and LC could be explained by the blood mediator using the medflex package in R. All statistical analyses were performed using R 4.1.1 software. A two-sided *p*-value < 0.05 was used for this study and considered statistically significant.

## 3. Results

### 3.1. Characteristics of the Participants

After matching the sex and age of the cases and controls, a total of 124 participants were enrolled (including 62 cases and 62 controls). The characteristics of the participants with LC and the controls are shown in Table 1. As expected, the participants showed similar characteristics. To broadly explore the distribution of blood indexes between those with LC and the controls, 10 blood indexes were detected, including white blood cell count (WBC), neutrophil count (NEUT), lymphocyte count (LYMPH), monocyte count (MONO), platelet count (PLT), eosinophil count (EOS), NLR, PLR, neutrophil monocyte ratio (NMR), and lymphocyte monocyte ratio (LMR). Compared with the control group, the LC group had higher WBC, MONO, PLT, NLR, PLR, and NMR values (all *p* < 0.05) and lower LYMPH and LMR values (*p* < 0.05).

### 3.2. Lipid Profiling Grouped by Lipid Structure and Risk of LC

Representative chromatograms of QC and case and control lipids in positive and negative ion mode are shown in Figures S1–S3. The results of unsupervised PCA are shown in Figure S4. A high experiment quality with a large degree of QC clustering was observed in unsupervised PCA. Apparent differences in grouping between the LC and control groups are shown in the PCA score scatter plots for the negative (Figure S4A) and positive (Figure S4B) ionization modes. A total of 605 lipid species spanning 20 individual lipid classes were identified in the plasma lipidome from 124 subjects, which is shown in Figure S4C. A volcano plot representing the levels of the lipids that were upregulated or downregulated in patients with LC compared with the control group is shown in Figure S4D.

**Figure 1:**
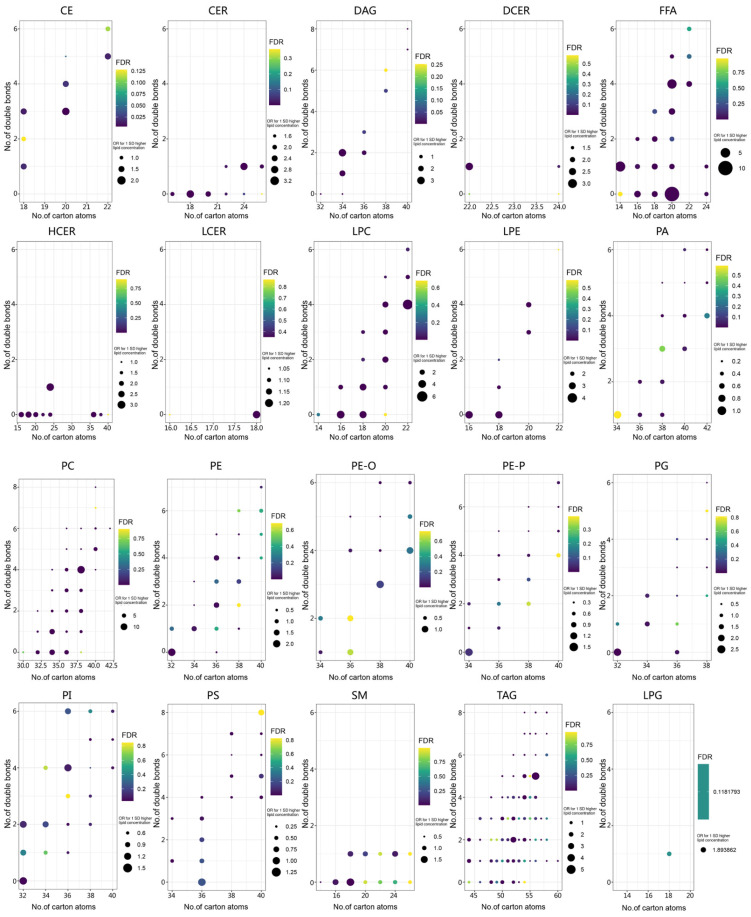
Association of fatty acid chain length and saturation level with LC risk by different lipid classes or subclasses through multivariable-adjusted conditional logistic regression. The *x*-axis denotes the total number of acyl-chain carbon atoms. The *y*-axis denotes the number of double bonds in the acyl chain. Circle size refers to the significance level indicated by *p* value and circle color refers to the magnitude of the OR per 1-SD (log scale) increment.

An overview of the relationship between 20 lipid subclasses and LC risk is described in Figure S5. Cholesteryl esters (CE), ceramides (CER), DCER, free fatty acid (FFA), hexosylceramide (HCER), PC, LPC, and lysophosphatidylethanolamine (LPE) were positively correlated with LC, while phosphates (PA), PE (O), PE (P) and phosphoserines (PS) were negatively correlated. DAG, LCER, LPG, PE, PG, PI, SM, and TAG were not significantly associated with LC. To clarify the potential biological mechanisms of each lipid with LC, the effect of the lipids based on their chemical structure was obtained using a multivariate conditional logistic regression model which is visualized in Figure 1. The ORs for the individual lipids and their FDR values are plotted in a two-dimensional graph defined by the number of carbon atoms (*x*-axis) and the number of double bonds (*y*-axis) in the acyl chain (detailed Supplemental Table S3 Sheet 4 includes the exact OR and FDR-corrected *p*-values). CE, CER, DCER, FFA, HCER, LPC, LPE, and PC positively related to LC risk, whereas PA, PE, PI and PS were related to lower odds of LC. Higher carbon atoms with DCER, PE, and PI presented a significant, negative correlation with LC. However, we did not find any clear correlation between the parity of the number of double bonds and the carbon atoms with LC risk. Compared with the FFA of unsaturated double bonds, the risk of saturated double bonds positively correlated with LC risk.

Moreover, the n-3 PUFA score and n-6 PUFA scores in the acyl chain were calculated, and point estimates of all lipids revealed an inverse association between LC risk and n-3 PUFA scores and the n-6/n-3 ratios obtained from all the lipid species, while there was a positive association between n-6 PUFA scores and LC risk. These results are shown in Table 2. Significant and negative associations between n-3 PUFA scores and LC risk were observed in DAG, PA, PE, PS, and TAG classes, whereas significant and positive associations were observed between n-6 PUFA scores and LC risk in FFA, LPC, LPE and TAG classes (all *p* < 0.05).

### 3.3. Screening for Differential Lipids and the Risk of LC

**Figure 2:**
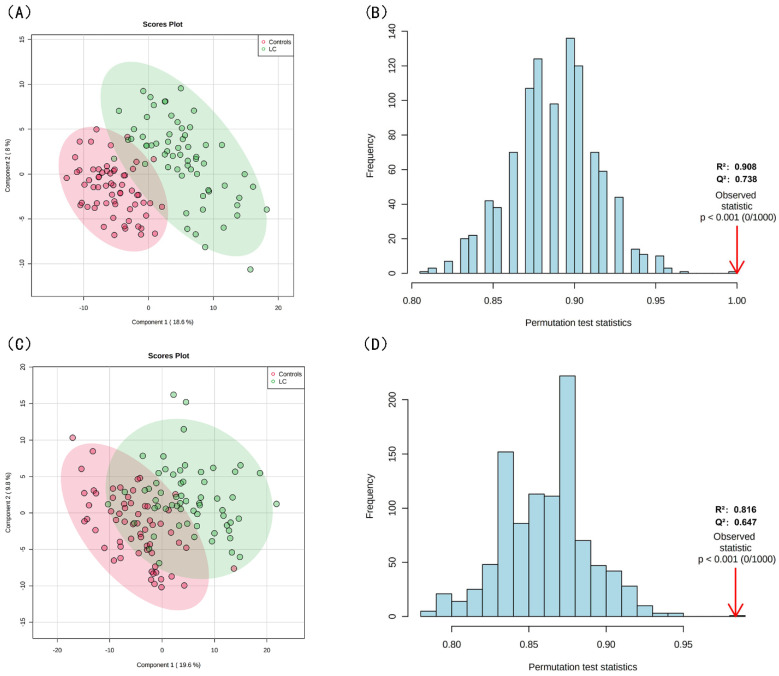
Results of PLS−DA (**A**) PLS−DA score plot of neg. (**B**) PLS−DA score plot of pos. (**C**) Validation of PLS−DA neg by permutation test performed with 1000 random permutations test. (**D**) Validation of PLS−DA pos by permutation test performed with 1000 random permutations test.

**Figure 3:**
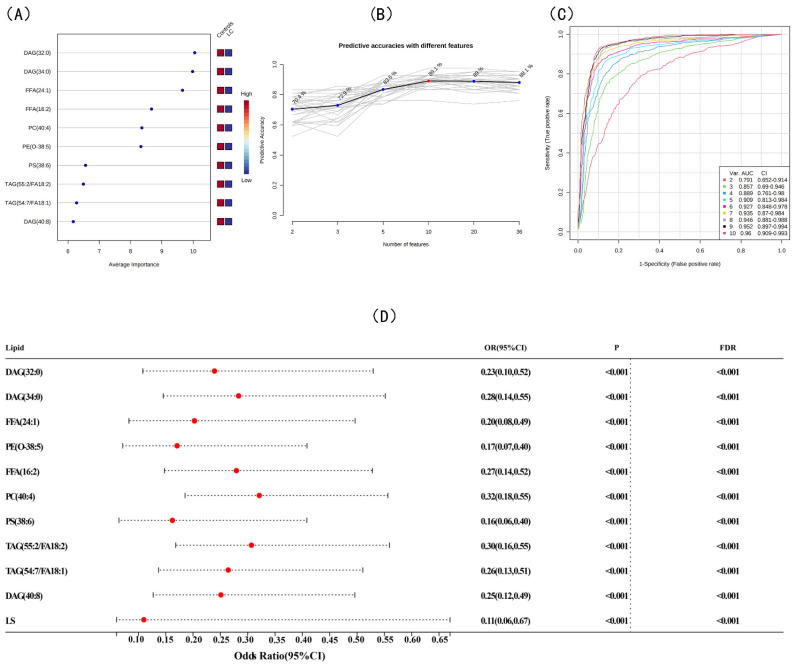
Selection of differential lipid between LC and controls. (**A**) Lipidomics metabolic biomarkers of RF classification. (**B**) Plot of the predictive accuracy of biomarker models (RF). (**C**) ROC curve analysis for biomarkers. (**D**) Forest plot.

PLS-DA, OPLS-DA, and sPLS-DA were used to identify differences in lipid profiles between the LC and control groups in positive and negative modes (Figure 2A,C and Figure S6A–H). Compared with OPLS-DA and sPLS-DA, there were remarkable separations with the performance of R^2^ = 0.816 and Q^2^ = 0.647 in the positive mode and R^2^ = 0.908 and Q^2^ = 0.738 in the negative mode of PLS-DA for the LC and control groups (Figure 2B,D). After combining the FC, *t*-tests, and VIP from PLS-DA, 36 differential lipid species were extracted with an FC > 2.0, an FDR-corrected *p*-value < 0.05, and a VIP value > 1.5. The association between the 36 differential lipids and LC risk are shown in Figure S7. When applying 36 differential lipids in KEGG, the pathways of GP metabolism, glycosylphosphatidylinositol-anchor biosynthesis, and GL metabolism were detected (Figure S6I). SVM and random forest algorithms were used to further obtain significant lipid biomarkers between the LC and control groups, and the random forest algorithm had a better predictive accuracy of the biomarker model (Figure 3A,B and Figure S8). A panel of 10 lipid biomarkers was identified by the random forest algorithm and included DAG (34:0), DAG (32:0), FFA (16:2), FFA (24:1), PE (O-38:5), PC (40:4), PS (38:6), TAG (55:2/FA 18:2), TAG (54:7/FA 18:1), and DAG (40:8). Lower odds were observed between DAG (34:0), DAG (32:0), FFA (16:2), FFA (24:1), PE (O-38:5), PC (40:4), PS (38:6), TAG (55:2/FA 18:2), TAG (54:7/FA 18:1), DAG (40:8) and LC risk in the multivariate conditional logistic regression (OR = 0.23, 95% CI: 0.10–0.52; OR = 0.28, 95% CI: 0.14–0.55; OR = 0.20, 95% CI: 0.08–0.49; OR = 0.17, 95% CI: 0.07–0.40; OR = 0.27, 95% CI: 0.14–0.52; OR = 0.32, 95% CI: 0.18–0.55; OR = 0.16, 95% CI: 0.06–0.40; OR = 0.30, 95% CI: 0.16–0.55; OR = 0.26, 95% CI: 0.13–0.51; and OR = 0.25, 95% CI: 0.12–0.49, respectively) (Figure 3D). Notably, there was a high predictive performance of lipid biomarkers with AUC = 0.909 (95% CI: 0.813–0.984) for 5 lipid species and AUC = 0.96 (95% CI: 0.909–0.993) for 10 lipid species (Figure 3C). Moreover, an LS based on 10 lipid biomarkers was calculated, and the association between LS and LC risk was investigated (OR = 0.11, 95% CI: 0.06–0.67). As shown in Figure S9, a higher LS was observed in the control group compared with the LC group and showed close and high predictive efficiency (0.96, 95% CI: 0.92–1.00). We also divided the 62 patients into two groups, including 28 in the early-stage group (stages 0 and I) and 34 in the intermediate- and advanced-stage groups (stages II–IV). Similarly, we screened for nine differential lipids between the early and intermediate to late groups and then showed the association between lipid signatures and lung cancer via forest plots (Figure S10).

### 3.4. Mediation Effects for Blood Indexes between Lipid Biomarkers and LC

Figure S9 shows LS and ROC curve analyses for the LC and control groups. Next, the association between blood indexes and lipid biomarkers was investigated (Figure S11). Generally, LMR and LYMPH showed a positive correlation with lipid biomarkers and LS, whereas PLR, NLR, and PLT were negatively associated with lipid biomarkers. In addition, MONO, NEUT, and WBC were negatively correlated with some markers, including LS, TAG (55:2/FA 18:2), TAG (54:7/FA 18:1), PE (O-38:5), PC (40:4), PS (38:6), and DAG (40:8).

A mediation analysis was performed to test whether the observed associations between LS and LC could be explained by a blood mediator. As shown in Table 3, LS had a partial, indirect effect on LS through LMR, LYMPH, MONO, NLR, and PLR with a matched 95% CI that excluded zero. The LMR, LYMPH, MONO, NLR, and PLR mediated proportions were 2.87%, 1.89%, 2.03%, 5.04%, and 2.95%, respectively.

## 4. Discussion

In the current study, we used targeted HPLC-MS/MS lipidomics and multiple statistical analyses to identify and quantify potentially differential lipid molecules associated with LC and reveal the specific relationships between different lipid structures and LC. Collectively, these findings capture the characteristic metabolic fingerprints of LC patients and elucidate the role of inflammatory mediators between lipids and LC, which adds value to recent studies on lipid metabolic reprogramming. To our knowledge, this is the first relatively comprehensive lipidomics study to explore lipid structural profiles and LC risk. In addition, this study innovatively used inflammatory indicators in blood as mediators to explore the relationships between different lipid molecules and LC.

One ST (CE), one FA (FFA), three SPs (CER, DCER, and HCER), and three GPs (LPC, LPE, and PC) were directly associated with LC risk, while the other four GPs (PA, PE, PI, and PS) were inversely associated with LC risk. The dysregulation of CE metabolism has been demonstrated in many tumor biomarker studies [19,20,21], including studies on LC [22]. A pilot study identified two lipid markers that distinguish squamous cell LC from high-risk individuals with high sensitivity, specificity, and accuracy, including CE (C 18:2) [23]. A study in Germany showed a significant accumulation of free cholesterol and cholesteryl esters within lung tumor tissue, and based on reports of elevated cholesterol levels in cancer cells, strategies to reduce cholesterol synthesis have been suggested as an anti-tumor strategy [24]. SPs are bioactive lipids that are involved in the modulation of cell survival, proliferation, and inflammatory responses, and the SphK/S1P/S1PR (S1P) pathway drives many anti-apoptotic and proliferative processes [25]. The disruption of SP metabolism has been associated with the pathogenesis of LC [6,26]. From the perspective of conversion, lipid-based multi-biomarker panels may capture information on the common etiological mechanisms of LC.

We identified 10 lipids as a fingerprint of patients with LC, and the results showed that 10 promising lipid biomarkers had an AUC range of 0.960. Changes in phospholipid metabolism significantly impact membrane structure, thereby affecting its function, altering key cellular signaling pathways (e.g., cell proliferation and survival), and promoting tumorigenesis [27]. For instance, altered PC and PE membrane content, phospholipid metabolite levels, and FA profiles are commonly recognized as indicators of carcinoma development and progression [28]. PC and PE are phospholipids (crucial components of the cell membrane), and their quantities have been altered in various malignancies [29]. PC could govern cancer cell death, and the blood levels of various PCs were significantly lower in LC patients [30]. PE participates in cell signaling and may control cellular growth and death programs. PE serum levels were higher in participants with malignant LC and decreased after surgical excision of the malignant nodules [31]. In a nested case control study conducted in China, PC and PE-O showed significantly different levels between the LC and control groups and were negatively associated with LC risk [32]. DAG plays a lipid second messenger role used to transduce signals. Few studies have discovered an association between DAG and LC; however, the induction of LC by combining DAG kinase brings etiological implications [33,34]. FFA, which acts as a substrate for cell membrane structures, has been considered an independent risk factor for cancer [35]. The association between FA and LC has been extensively described [36,37]. A previous review discussed the role of fatty acids and their lipid mediators in the apoptosis of cancer cells [38]. Inconsistent study results may be partly due to differences in cancer type, study design, population, sample size, and confounding elimination.

There are findings in our study that deserve particular attention. The association of lipids with LC risk can differ based on acyl chain length and the unsaturation degree; therefore, the lipids were further analyzed based on their carbon atom and double bond numbers. Our results partially corroborated previous observations [39] that DCER, PE, and PI with longer acyl chains were associated with a lower risk of LC. Chen et al. demonstrated that short carbon chain C2-ceramide can effectively sensitize PTX-induced senescence of H1299 cells via p21waf1/cip1- and p16ink4-independent pathways [39]. A previous study [31] suggested that long-chain PE is a potential diagnostic marker for LC. The overexpression of phosphatidylethanolamine-binding protein 4 (PEBP4) in LC regulates tumor progression, invasion, and metastatic potential, which may be partly due to an increase in PEs that act as agonists of PEBP and mediate signal transduction [40]. The relationship between FFA and LC varied at differing degrees of unsaturation. Our results, as well as previous studies [41], support the hypothesis that SFA may be significant in the etiology of LC and deterministic in its development. Saturated lipids are less susceptible to lipid peroxidation, which may protect cancer cells from lipid peroxidation-mediated cell death, and it also alters membrane dynamics and affects the uptake and efficacy of chemotherapeutics [12]. PUFA was found to be important for maintaining cellular function and internal environmental homeostasis, including signal transduction, cell growth, differentiation, and viability [37]. In particular, the levels of n-3 and n-6 PUFAs play an important role in LC risk and progression. Also, n-3 PUFAs contain docosahexaenoic acid (DHA), eicosapentaenoic acid (EPA), and alpha-linolenic acid (ALA). A previous study reported that DHA-PC and EPA-PC significantly inhibited orthotopic tumor growth and lung metastasis, via the activation of PPARγ and the downregulation of the NF-κB pathway to control tumor growth and metastasis [42]. Another study [36] used genome-wide association study (GWAS) data from a Mendelian randomization (MR) approach to demonstrate that n-3 PUFA is a causal protective factor for LC, which is similar to our results. In addition, a mouse experiment [43] showed that mice that were fed a diet rich in n-6 PUFA had significantly increased proliferation, angiogenic and pro-inflammatory markers, and decreased expression of pro-apoptotic proteins in their tumors. Nevertheless, the association between n-6 PUFA, n6/n3 ratios, and LC risk was not found in our study.

In terms of the causal association between lipids and LC, it is plausible that the mediation effects of inflammatory mediators can be partially explained. Previous studies [44,45] have discovered an association between inflammatory mediators and lipid metabolites. Qian et al. [46] demonstrated that a key mechanism involved in inflammatory states is GP metabolism, which raises the possibility that phospholipids may act as inflammatory mediators. The polyunsaturated alkenyl-linked fatty acids found in PE(P) and PE(O), together known as plasmalogens, are essential for the storage of precursors as inflammatory mediators, the control of membrane fluidity, and anti-oxidation [47]. LPC plays an important role in mediating inflammation [48] and endothelial cell activation [49]. In addition, systemic inflammatory biomarkers relate to the occurrence and progression of cancer, including LC [50,51,52]. A prospective UK Biobank cohort recruited 440,000 participants and assessed the associations between systemic inflammation markers and risks for 17 cancer sites and revealed that inflammation markers could serve as biomarkers of cancer [53]. In the current study, it was revealed that LMR, LYMPH, MONO, NLR, and PLR mediated 2.87%, 1.89%, 2.03%, 5.04%, and 2.95%, respectively, of associations between LS and LC risk. Nevertheless, future research must still determine the precise processes underlying the detected connections.

The major strengths in our current study lie in its analytical approaches and well-characterized study design. Our targeted lipidomics approach was constructed upon HPLC-MS/MS, which allowed for the explicit identification and relative quantification of plasma lipids. Two univariate methods, three multivariate methods, and two machine learning methods were used to select differential lipid molecules, and their similarities and differences were compared. To our knowledge, the present study is the first to explore the relationship between lipids, blood inflammatory markers, and LC risk, and it provides a new perspective for future studies. However, the study has several limitations. Selection bias may be present in any hospital-based case control study, but all subjects were recruited according to strict criteria, which may minimize selection bias. We also did not further explore the classification and staging of LC. Therefore, we will consider expanding the study sample, refining the subgroups, and staging of the study population in future studies.

Finally, current immunotherapies have shown remarkable effects for controlling cancer, with the PD-1/PD-L1 axis being one of the most important and well-studied checkpoint pathways in cancer immunity [54]. Immune-related adverse events were found to be closely related to the mechanism by which PD-1/PD-L1 antibodies restarted anti-cancer immune attacks in a lung cancer study by Sun et al. [55]. The specific role of lipids in the regulation of the PD-1/PD-L1 axis was revealed by Yang et al. [54]. Lipids are key metabolic switches in the immune response [54,56]. The link between lipids and cancer immunity provides an opportunity for future studies to use lipids as biomarkers to evaluate cancer immune responses.

## 5. Conclusions

In summary, the current study provides a comprehensive analysis of the plasma levels of 605 lipids. The findings of this study deepen our knowledge of the pathophysiological mechanisms of LC, highlight the importance of detailed studies on structural differences between various species of lipids in LC research, reveal relationships between different lipid subclass characteristics and LC risk, identify 10 lipid metabolites as potential novel biomarkers of LC risk, and explore associations between lipids and blood inflammatory indicators. In addition, clearly altered lipids were noted to be related to GP metabolism and alpha-linolenic acid metabolism. Our results suggest that lipid profiling may provide novel tools for the research of LC and facilitate the advancement of disease diagnosis and treatment. Blood lipids are promising LC biomarkers that may lead to new treatment strategies.

## Figures and Tables

**Table 1:** Characteristics and blood parameters of participants between LC and control group ^1^.

Variables	N	LC	Controls	*p* ^2^
Sex				
Male	80	40 (64.5%)	40 (64.5%)	
Female	44	22 (35.5%)	22 (35.5%)	
Age (years)				
≤54	64	30 (48.4%)	34 (54.8%)	
>54	60	32 (51.6%)	28 (45.2%)	
BMI(kg/m²)				0.853
≤24	93	46 (74.2%)	47 (75.8%)	
>24	31	16 (25.8%)	15 (24.2%)	
WBC (×10^9^/L)	124	8.88 (5.43,12.33)	6.32 (5.97,6.68)	0.018
NEUT (×10^9^/L)	124	4.73 (4.21,5.24)	3.54 (3.28,3.80)	<0.001
LYMPH (×10^9^/L)	124	1.67 (1.51,1.84)	2.17 (2.03,2.31)	<0.001
MONO (×10^9^/L)	124	0.59 (0.44,0.74)	0.39 (0.37,0.43)	0.002
PLT (×10^9^/L)	124	284 (257,310)	239 (227,251)	0.010
EOS (×10^9^/L)	124	0.21 (0.13,0.29)	0.17 (0.14,0.20)	0.384
NLR	124	3.26 (2.76,3.76)	1.69 (1.55,1.82)	<0.001
PLR	124	199 (166,233)	116 (107,125)	<0.001
NMR	124	9.86 (8.73,10.99)	9.21 (8.56,9.87)	<0.001
LMR	124	3.65 (3.13,4.18)	5.67 (5.33,6.02)	<0.001

^1^ Variables are mean ± SD for continuous variables and n (%) for categorial variables. WBC: white blood cell count; NEUT: neutrophil count; LYMPH: lymphocyte count; MONO: monocyte count; PLT: platelet count; EOS: eosinophil count; NLR: neutrophil lymphocyte ratio; PLR: platelet lymphocyte ratio; NMR: neutrophil monocyte ratio; LMR: lymphocyte monocyte ratio. The ^2^ *p* values were estimated by univariable conditional logistic regression except for matching variables (sex and age).

**Table 2:** Relationship between polyunsaturated fatty acids among subclass and the risk of LC.

	OR(95%CI) *	*p*	FDR
n-6 PUFA(all)	1.00 (0.99,1.02)	0.260	0.484
n-3 PUFA(all)	0.97 (0.96,0.98)	<0.001	0.001
n6/n3 ratio(all)	0.96 (0.90,1.04)	0.382	0.574
CE			
n-6 PUFA	1.27 (1.03,1.56)	0.024	0.072
n-3 PUFA	1.09 (0.98,1.22)	0.099	0.215
n6/n3 ratio	1.06 (0.88,1.29)	0.494	0.600
DAG			
n-6 PUFA	1.13 (0.99,1.30)	0.065	0.170
n-3 PUFA	0.53 (0.39,0.73)	<0.001	0.001
n6/n3 ratio	0.98 (0.94,1.01)	0.334	0.556
FFA			
n-6 PUFA	2.11 (1.45,3.09)	<0.001	0.001
n-3 PUFA	0.95 (0.83,1.07)	0.438	0.600
n6/n3 ratio	0.99 (0.97,1.01)	0.573	0.638
LPC			
n-6 PUFA	1.69 (1.28,2.25)	<0.001	0.001
n-3 PUFA	1.12 (1.00,1.25)	0.043	0.121
n6/n3 ratio	0.99 (0.97,1.01)	0.562	0.638
LPE			
n-6 PUFA	1.67 (1.23,2.26)	<0.001	0.003
n-3 PUFA	1.13 (0.80,1.61)	0.466	0.600
n6/n3 ratio	1.00 (0.98,1.01)	0.945	0.945
PA			
n-6 PUFA	0.81 (0.72,0.91)	<0.001	0.003
n-3 PUFA	0.55 (0.39,0.77)	<0.001	0.002
n6/n3 ratio	1.05 (0.93,1.19)	0.360	0.561
PC			
n-6 PUFA	1.03 (0.99,1.07)	0.085	0.196
n-3 PUFA	1.01 (0.97,1.05)	0.508	0.600
n6/n3 ratio	0.88 (0.77,1.02)	0.114	0.234
PE			
n-6 PUFA	0.96 (0.93,1.00)	0.079	0.194
n-3 PUFA	0.87 (0.81,0.93)	<0.001	0.001
n6/n3 ratio	0.99 (0.96,1.01)	0.494	0.600
PG			
n-6 PUFA	0.95 (0.87,1.04)	0.281	0.498
PI			
n-6 PUFA	0.99 (0.92,1.06)	0.831	0.853
n-3 PUFA	0.93 (0.84,1.03)	0.177	0.346
n6/n3 ratio	0.99 (0.98,1.00)	0.476	0.600
PS			
n-6 PUFA	0.85 (0.78,0.92)	<0.001	0.001
n-3 PUFA	0.70 (0.58,0.84)	<0.001	0.001
n6/n3 ratio	1.02 (0.97,1.06)	0.342	0.556
TAG			
n-6 PUFA	1.09 (1.03,1.14)	<0.001	0.002
n-3 PUFA	0.94 (0.92,0.97)	<0.001	0.001
n6/n3 ratio	0.99 (0.95,1.02)	0.620	0.672

* Adjusted for sex, age, and BMI.

**Table 3:** Mediation of blood parameters for the associations between LS and risk of LC.

Effects	Β (95%CI)	OR (95%CI)	*p*
Mediation factor = LMR			
Direct effect(LS→LC)	−2.09 (−3.34,−0.85)	0.12 (0.03,0.43)	<0.001
Indirect effect(LMR→LS→LC)	−0.24 (−0.41,−0.07)	0.79 (0.66,0.93)	0.005
	Proportion mediated = 2.87%		
Mediation factor = LYMPH			
Direct effect(LS→LC)	−2.05 (−2.95,−1.13)	0.13 (0.05,0.32)	<0.001
Indirect effect(LYMPH→LS→LC)	−0.14 (−0.26,−0.03)	0.87 (0.77,0.97)	0.014
	Proportion mediated = 1.89%		
Mediation factor = PLR			
Direct effect(LS→LC)	−1.99 (−2.94,−1.05)	0.14 (0.05,0.35)	<0.001
Indirect effect(PLR→LS→LC)	−0.14 (−3.09,−1.18)	0.87 (0.78,0.97)	0.013
	Proportion mediated = 2.03%		
Mediation factor = NLR			
Direct effect(LS→LC)	−1.68 (−2.62,−0.74)	0.19 (0.07,0.47)	<0.001
Indirect effect(NLR→LS→LC)	−0.26 (−2.85,−1.04)	0.77 (0.64,0.91)	0.003
	Proportion mediated = 5.04%		
Mediation factor = PLT			
Direct effect(LS→LC)	−1.90 (−2.83,−0.97)	0.15 (0.06,0.37)	<0.001
Indirect effect(PLT→LS→LC)	−0.19 (−0.32,−0.07)	0.83 (0.73,0.93)	0.002
	Proportion mediated = 2.95%		

Fully model was adjusted for sex, age, and BMI. LC: lung cancer; LS: lipid score; LMR: lymphocyte monocyte ratio; LYMPH: Lymphocyte count; PLR: platelet lymphocyte ratio; NLR: Neutrophil lymphocyte ratio; PLT: Platelet count.

## Data Availability

The datasets used in this study are available from the corresponding author on reasonable request.

## Supplementary Materials

The following supporting information can be downloaded at: https://www.mdpi.com/article/10.3390/jcm12051736/s1, Figure S1: Representative chromatograms of QC by lipidomics analyses in positive (A) and negative (B) modes; Figure S2: Representative chromatograms of case group by lipidomics analyses in positive (A) and negative (B) modes; Figure S3: Representative chromatograms of control group by lipidomics analyses in positive (A) and negative (B) modes; Figure S4: Lipid species identified by lipidomics; Figure S5: The association between 20 lipid subclasses and risk of LC; Figure S6: Multivariate analysis screening and comparison; Figure S7: The association between 36 differential lipids and risk of LC; Figure S8: Selection of SVM; Figure S9: Levels of LS in the LC and control group; Figure S10. Nine differential lipids between early and intermediate to advanced stage groups in association with LC; Figure S11: Heatmap of correlation between differential lipids and blood parameters; Table S1: HPLC-MS/MS parameters; Table S2: The parameters of the ESI source condition in positive and negative ion mode; Table S3: The detailed information of lipids; Supplementary Materials File S1. Internal Standards.
