# Supplementary material for: The Landscape of Lipid Metabolism in Lung Cancer: The Role of Structural Profiling
Source: J Clin Med. 2023 Feb 21;12(5):1736. doi: 10.3390/jcm12051736 (PMC10002589; doi:10.3390/jcm12051736)
Supplement: Supplementary file 1 [file jcm-12-01736-s001.zip › 2. Supplement Tables S1 and S2.pdf]

Supplementary Material-Table

List

1. Supplement Table S1 HPLC-MS/MS parameters
2. Supplement Table S2 The parameters of the ESI source condition in positive and negative ion mode

Table S1. HPLC-MS/MS parameters

Instrument	Sciex QTRAP 4500 with HPLC system
mobile phases A	95% ACN (working liquid) and mass spectrometry water (column washing liquid)
mobile phases B	50% ACN water (working liquid) and 100% ACN (washing column liquid)
Total flow rate	0.5 mL/min
Column	100 mm× 2.1 mm, 1.7μm
Column temperature	35℃
Gradient elution B	0min, 0.1%B; 10 min, 20% B; 11-13min, 98% B; 13.1 min, 0.1% B; 16min, stop.

Table S2. The parameters of the ESI source condition in positive and negative ion mode

parameters	pos	neg
IonSpray Voltage	5500 V	-4500 V
Curtain Gas	35 psi	35 psi
Collision Gas	Medium	Medium
Ion Source Gas 1	55 psi	55 psi
Ion Source Gas 2	55 psi	55 psi
Temperature（TEM）	350℃	350℃
Declustering Potential	80 V	-80 V
Entrance Potential	10 V	-10 V
Collision Cell Exit Potential	10 V	-10 V
