# Supplementary material for: The Landscape of Lipid Metabolism in Lung Cancer: The Role of Structural Profiling
Source: J Clin Med. 2023 Feb 21;12(5):1736. doi: 10.3390/jcm12051736 (PMC10002589; doi:10.3390/jcm12051736)
Supplement: Supplementary file 1 [file jcm-12-01736-s001.zip › 4. Supplementary Materials File S1. Internal Standards.pdf]

Certificate of Analysis

Product Identification

Name: Internal Standards Kit for Lipidyzer™ Platform
Part number: 5040156
Lot number: LPISTDKIT-100
Manufacture date: 15 DEC 2015
Expiration date: 15 DEC 2016

Part Number / Lot Number	Internal Standards	MW	Specified Concentration mg/ml	Actual Concentration +/-10%	Purity %
5040146 CERISTLPV-100	Ceramides (CER) Internal Standards for Lipidyzer™ Platform		0.02	0.02	100%
	dCER(d16:0)	546.97	0.02	0.02	

5040147 CHEISTLPV-100	Cholesterol Ester (CE) Internal Standards for Lipidyzer™ Platform		3.0	3.0	100%
	dCE(16:0)	631.62	0.15	0.13	
	dCE(16:1)	629.61	0.15	0.13	
	dCE(18:1)	657.64	0.60	0.57	
	dCE(18:2)	655.62	1.50	1.43	
	dCE(20:3)	681.64	0.15	0.15	
	dCE(20:4)	679.62	0.15	0.18	
	dCE(20:5)	677.61	0.15	0.18	
	dCE(22:6)	703.62	0.15	0.22	

5040148 DAGISTLPV-100	Diacylglycerol (DAG) Internal Standards for Lipidyzer™ Platform		0.025	0.025	100%
	dDAG(16:0/16:0)	577.56	0.005	0.004	
	dDAG(16:0/18:0)	605.59	0.005	0.005	
	dDAG(16:0/18:1)	603.57	0.005	0.006	
	dDAG(16:0/18:2)	601.56	0.005	0.005	
	dDAG(16:0/18:3)	599.54	0.00125	0.00135	
	dDAG(16:0/20:4)	625.56	0.00125	0.00150	
	dDAG(16:0/20:5)	623.54	0.00125	0.00145	
	dDAG(16:0/22:6)	649.56	0.00125	0.00160	

5040149 FFAISTLPV-100	Free Fatty Acids (FFA) Internal Standards for Lipidyzer™ Platform		0.10	0.10	100%
	dFFA(16:0)	265.48	0.05	0.05	
	dFFA(17:1)	268.48	0.05	0.05	

5040150 LPCISTLPV-100	Lysophosphatidylcholine (LPC) Internal Standards for Lipidyzer™ Platform		0.10	0.10	99.1%
	dLPC(16:0)	504.69	0.10	0.10	

Certificate of Analysis

Part Number / Lot Number	Internal Standards	MW	Specified Concentration mg/ml	Actual Concentration +/-10%	Purity %
5040151 LPEISTLPV-100	Lysophosphatidylethanolamine (LPE) Internal Standards for Lipidyzer™ Platform		0.05	0.05	100%
	dLPE(18:0)	486.64	0.05	0.05	
5040152 PCISTLPV-100	Phosphatidylcholine (PC) Internal Standards for Lipidyzer™ Platform		1.25	1.25	100%
	dPC(16:0/16:1)	740.60	0.0625	0.0575	
	dPC(16:0/18:1)	768.63	0.2500	0.2525	
	dPC(16:0/18:2)	766.62	0.2500	0.2550	
	dPC(16:0/18:3)	764.60	0.0625	0.0650	
	dPC(16:0/20:3)	792.63	0.0625	0.0725	
	dPC(16:0/20:4)	790.62	0.2500	0.2775	
	dPC(16:0/20:5)	788.60	0.0625	0.0700	
	dPC(16:0/22:4)	818.65	0.0625	0.0750	
	dPC(16:0/22:5)	816.63	0.0625	0.0775	
	dPC(16:0/22:6)	814.62	0.1250	0.1450	
5040153 PEISTLPV-100	Phosphatidylethanolamine (PE) Internal Standards for Lipidyzer™ Platform		0.05	0.05	100%
	dPE(18:0/18:1)	750.59	0.01	0.01	
	dPE(18:0/18:2)	748.58	0.01	0.01	
	dPE(18:0/18:3)	746.56	0.0025	0.0021	
	dPE(18:0/20:3)	774.59	0.0025	0.0027	
	dPE(18:0/20:4)	772.58	0.01	0.01	
	dPE(18:0/20:5)	770.56	0.0025	0.0022	
	dPE(18:0/22:5)	798.60	0.0025	0.0024	
	dPE(18:0/22:6)	796.58	0.01	0.01	
5040154 SMISTLPV-100	Sphingomyelin (SM) Internal Standards for Lipidyzer™ Platform		0.4	0.4	100%
	dSM(16:0)	709.61	0.1	0.1	
	dSM(18:1)	735.62	0.1	0.1	
	dSM(24:0)	821.73	0.1	0.1	
	dSM(24:1)	819.72	0.1	0.1	

Certificate of Analysis

Part Number / Lot Number	Internal Standards	MW	Specified Concentration mg/ml	Actual Concentration +/-10%	Purity %
5040155 TAGISTLPV-100	Triacylglycerol (TAG) Internal Standards for Lipidyzer™ Platform		0.70	0.70	100%
	dTAG50:1-FA16:0	841.81	0.14	0.13	
	dTAG52:1-FA18:0	869.84	0.14	0.14	
	dTAG52:2-FA18:1	867.82	0.14	0.14	
	dTAG52:3-FA18:2	865.80	0.14	0.14	
	dTAG52:4-FA18:3	863.79	0.035	0.040	
	dTAG54:4-FA20:3	891.82	0.035	0.040	
	dTAG54:5-FA20:4	889.80	0.035	0.040	
	dTAG56:7-FA22:6	913.80	0.035	0.038	

5040387 DCERISLPV-100	Dihydroceramides (DCER) Internal Standards for Lipidyzer™ Platform		0.004	0.004	100%
	dDCER(16:0)	548.99	0.004	0.004	

5040388 HCERISLPV-100	Hexosylceramides (HCER) Internal Standards for Lipidyzer™ Platform		0.03	0.03	100%
	dHCER(16:0)	709.11	0.03	0.03	

5040389 LCERISLPV-100	Lactosylceramide (LCER) Internal Standards for Lipidyzer™ Platform		0.03	0.03	100%
	dLCER(16:0)	871.25	0.03	0.03	

Quality Assurance Statement:

These standards have been manufactured using ISO9001 procedures. Concentrations are calculated gravimetrically, and balances are calibrated to NIST traceable standards in accordance with our ISO procedures. Purity from interferences is verified through mass spectrometric analysis. Solvents are minimum HPLC grade.

The information on this certificate has been reviewed and accurately reflects manufacturing and analysis data for the product lot specified.

Quality Assurance Associate: LC   Date: 29 DEC 2015

Support

Technical Support call 1 800-952-4716 or 877-740-2129 or E-mail: Support@sciex.com

This certificate may be obtained at: http://sciex.com

AB Sciex Pte. Ltd.
500 Old Connecticut Path
Framingham, MA 01701
U.S.A.
