# Supplementary material for: The Landscape of Lipid Metabolism in Lung Cancer: The Role of Structural Profiling
Source: J Clin Med. 2023 Feb 21;12(5):1736. doi: 10.3390/jcm12051736 (PMC10002589; doi:10.3390/jcm12051736)
Supplement: Supplementary file 1 [file jcm-12-01736-s001.zip › SupplementaryFigures.pdf]

Supplementary Material-Figure

List

1. **Supplement Figure S1**. Representative chromatograms of QC by lipidomics analyses in positive (A) and negative (B) modes.

2. **Supplement Figure S2**. Representative chromatograms of case group by lipidomics analyses in positive (A) and negative (B) modes.

3. **Supplement Figure S3**. Representative chromatograms of control group by lipidomics analyses in positive (A) and negative (B) modes.

4. **Supplement Figure S4**. Lipid species identified by lipidomics.
   (A) PCA score plot of NEG
   (B) PCA score plot of POS
   (C) Identification of lipid categories and counts.
   (D) volcano plot
5. **Supplement Figure S5**. The association between 20 lipid subclasses and risk of LC
6. **Supplement Figure S6**. Multivariate analysis screening and comparison.
   (A) OPLS-DA score plot of neg.
   (B) OPLS-DA score plot of pos.
   (C) Validation of OPLS-DA neg by permutation test performed with 1000 random permutations test.
   (D) Validation of OPLS-DA pos by permutation test performed with 1000 random permutations test.
   (E) sPLS-DA score plot of NEG.
   (F) sPLS-DA score plot of POS.
   (G)Plot of the performance of the sPLS-DA model(neg)
   (H) Plot of the performance of the sPLS-DA model(pos).
   (I) Summary of pathway analysis by Metaboanalyst online.

7. **Supplement Figure S7**. The association between 36 differential lipids and risk of LC

8. **Supplement Figure S8**. Selection of SVM
   (A) lipidomics metabolic biomarkers of SVM classification.
   (B) Plot of the predictive accuracy of biomarker models (SVM)

9. **Supplement Figure S9**. levels of LS in the LC and control group
   (A) Box plot.
   (B) ROC curve analysis of LS

10. **Supplement Figure S10**. Nine differential lipids between early and intermediate to advanced stage groups in association with LC.

11. **Supplement Figure S11**. Heatmap of correlation between differential lipids and blood parameters

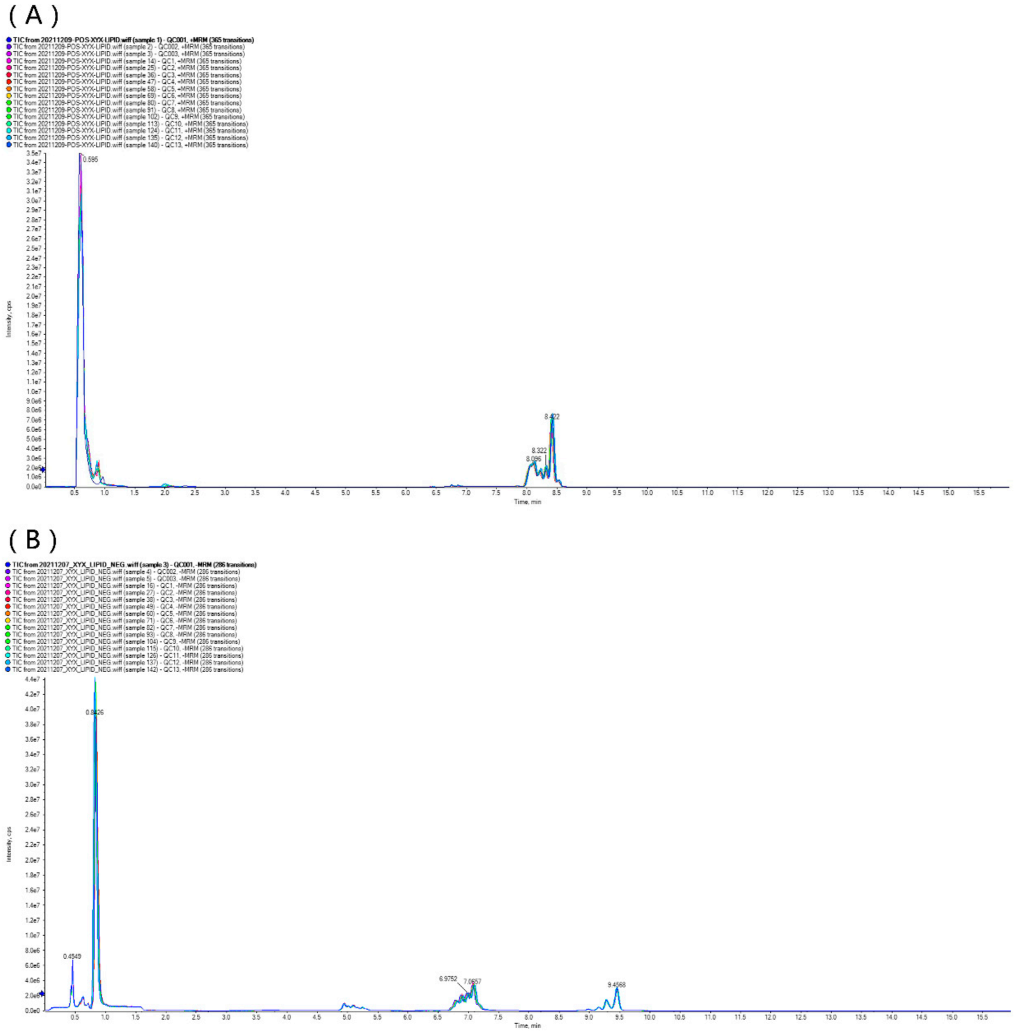

Supplement Figure S1. Representative chromatograms of QC by lipidomics analyses in positive (A) and negative (B) modes.

(A)

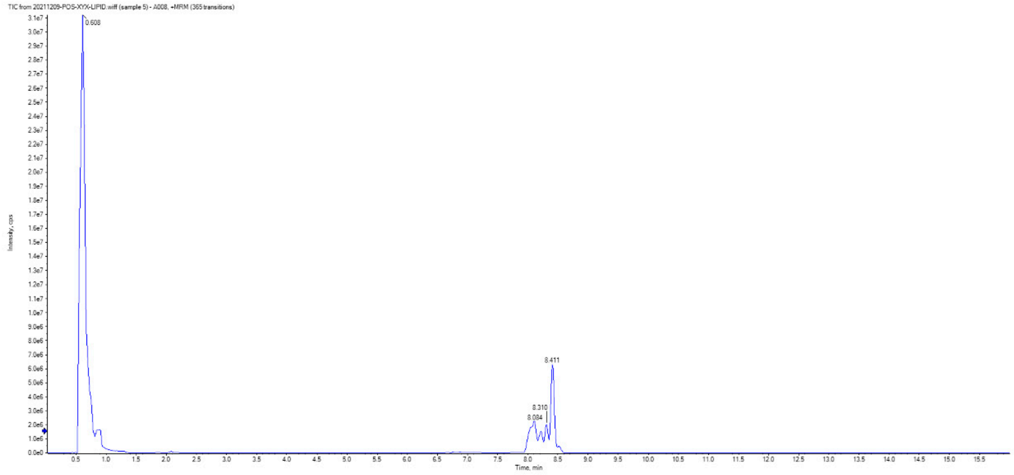

(B)

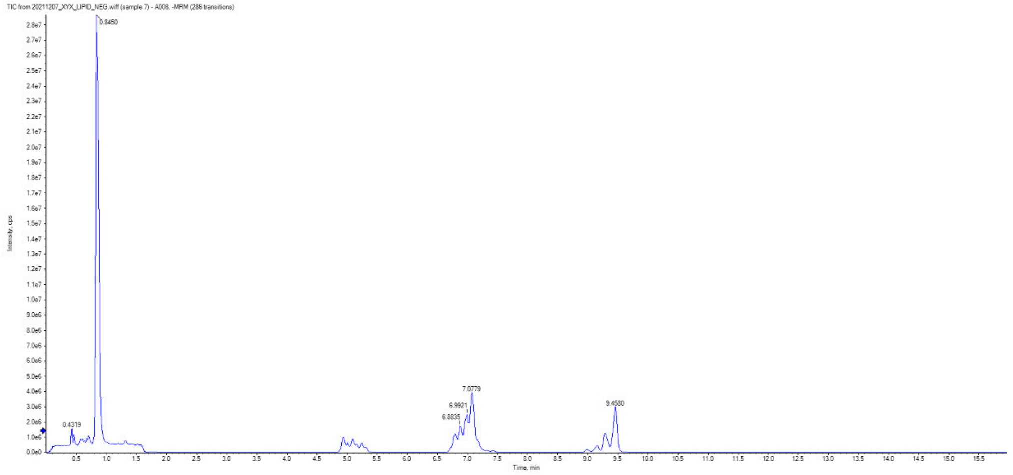

Supplement Figure S2. Representative chromatograms of case group by lipidomics analyses in positive (A) and negative (B) modes.

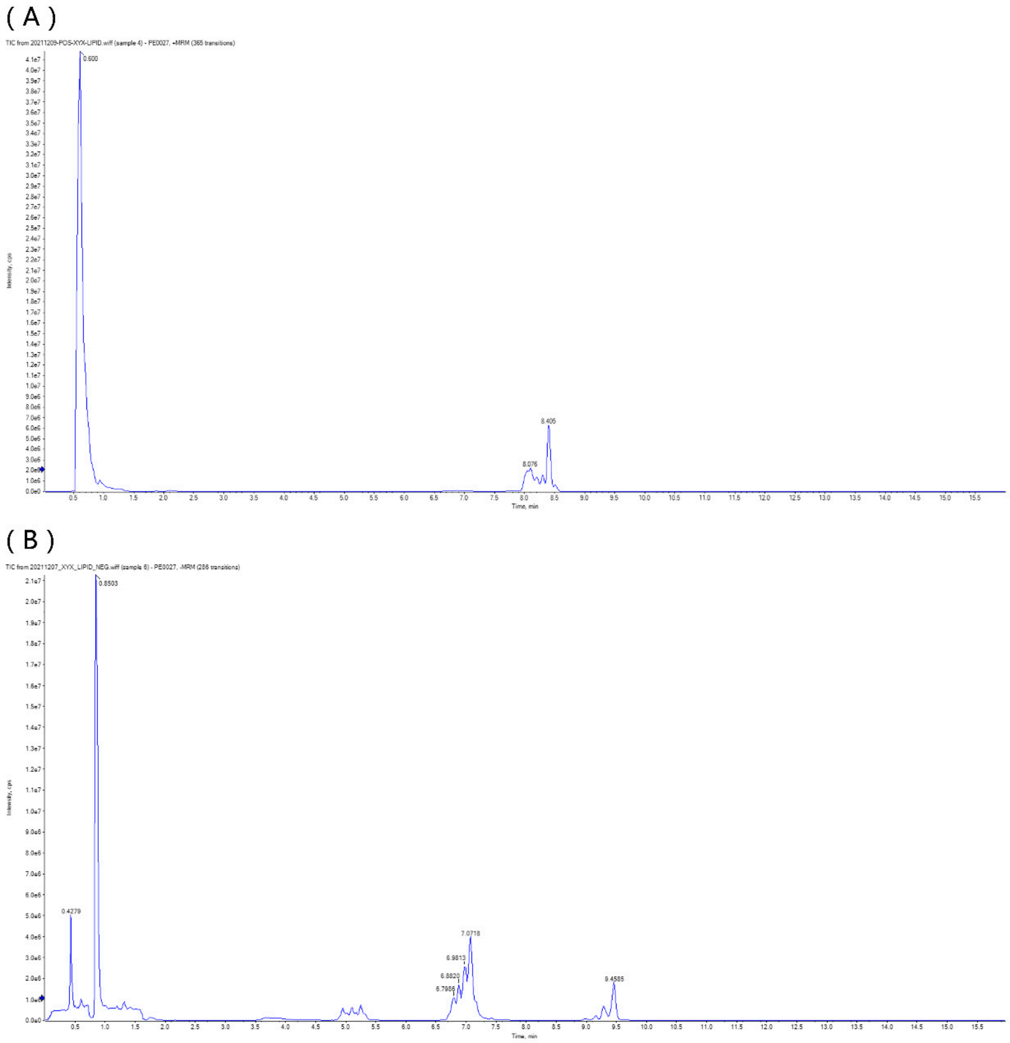

Supplement Figure S3. Representative chromatograms of control group by lipidomics analyses in positive (A) and negative (B) modes.

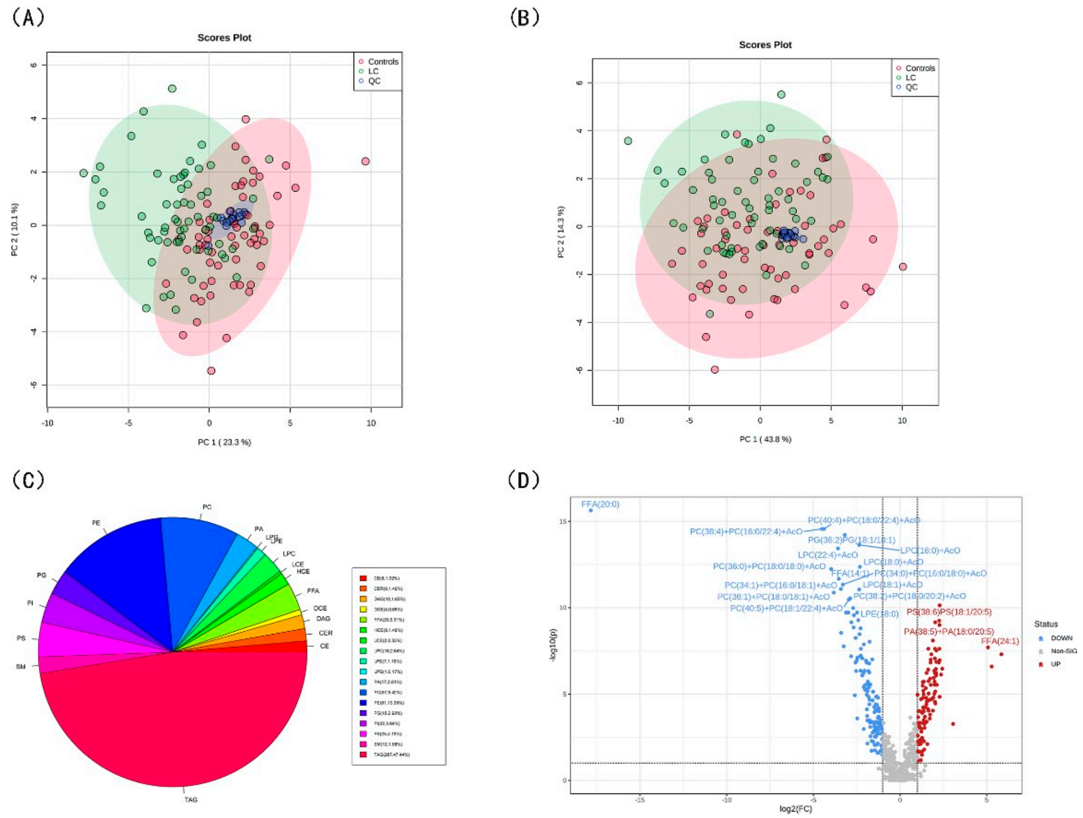

Supplement Figure S4. Lipid species identified by lipidomics.

(A) PCA score plot of NEG (B) PCA score plot of POS (C) Identification of lipid categories and counts. (D) volcano plot

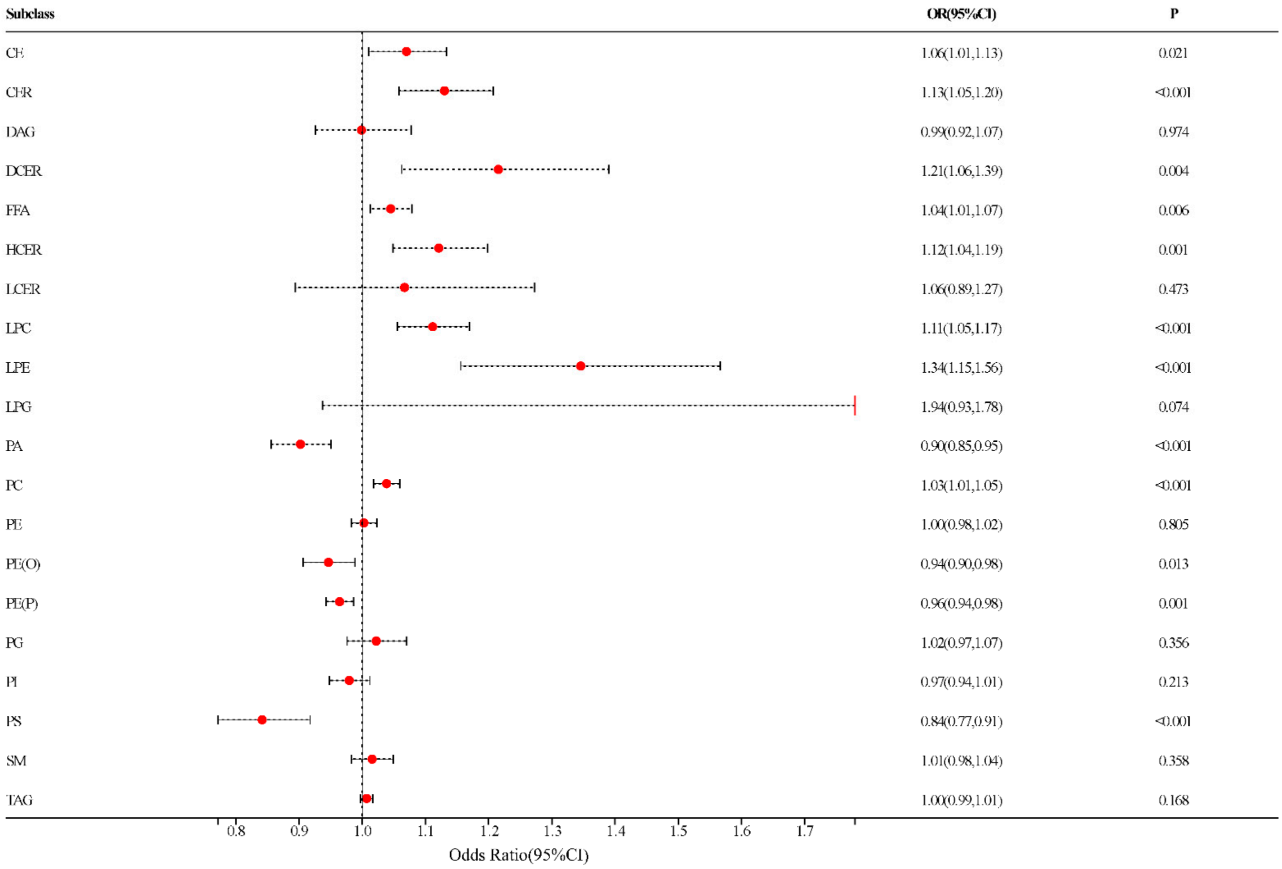

Supplement Figure S5. The association between 20 lipid subclasses and risk of LC

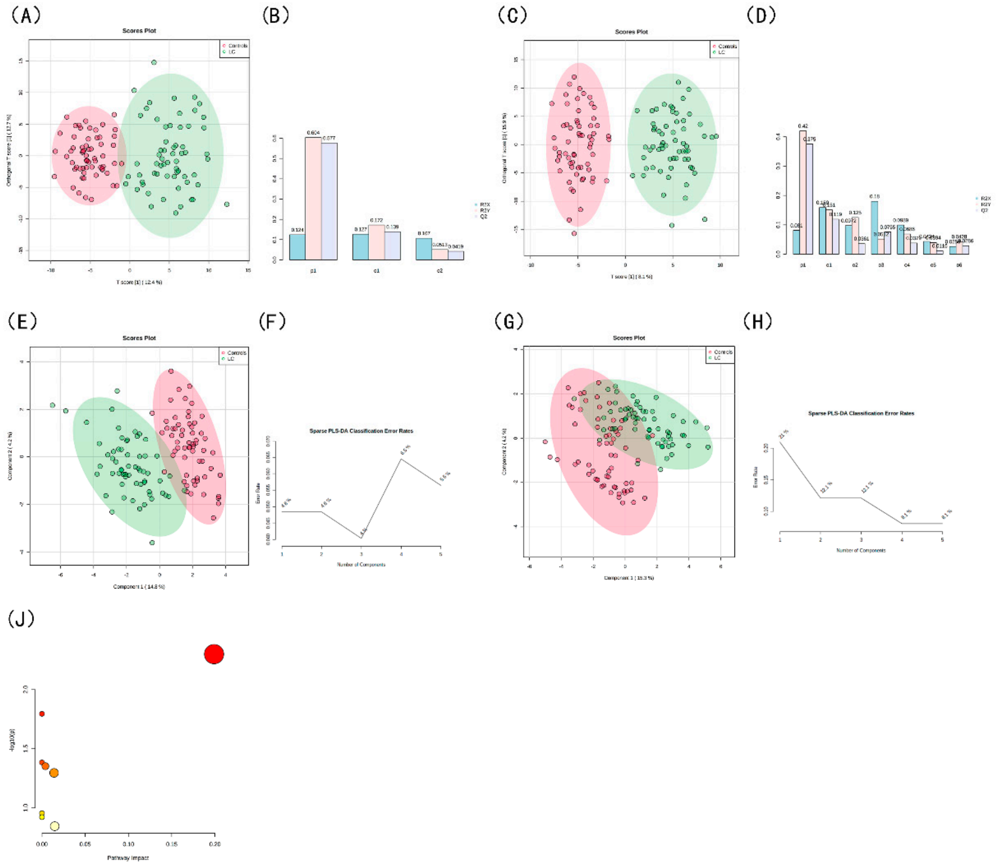

Supplement Figure S6. Multivariate analysis screening and comparison.
(A) OPLS-DA score plot of neg. (B) OPLS-DA score plot of pos. (C) Validation of OPLS-DA neg by permutation test performed with 1000 random permutations test. (D) Validation of OPLS-DA pos by permutation test performed with 1000 random permutations test. (E) sPLS-DA score plot of NEG. (F) sPLS-DA score plot of POS. (G)Plot of the performance of the sPLS-DA model(neg)
(H) Plot of the performance of the sPLS-DA model(pos). (I) Summary of pathway analysis by Metaboanalyst online.

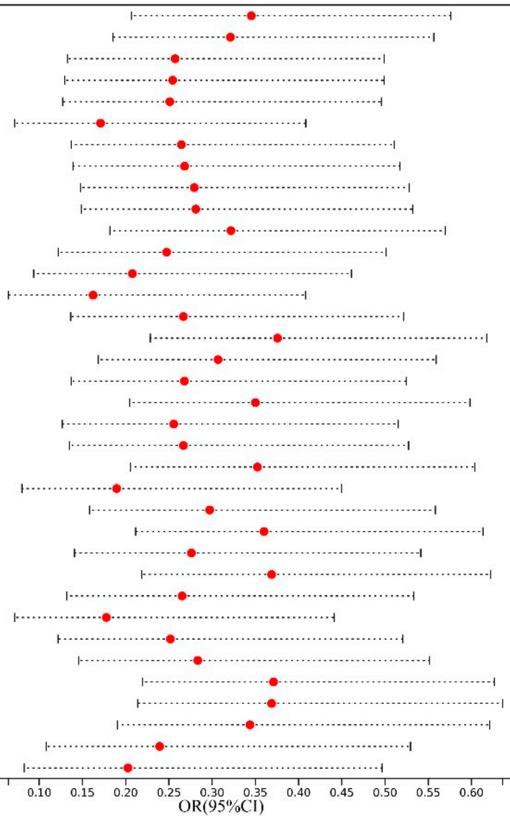

Lipid	OR(95%CL)	P	FDR
TAG(56:9/FA22:6)	0.34(0.20,0.57)	<0.001	<0.001
PC(40:4)	0.32(0.18,0.55)	<0.001	<0.001
TAG(60:12/FA22:6)	0.25(0.13,0.49)	<0.001	<0.001
TAG(54:8/FA22:6)	0.25(0.12,0.49)	<0.001	<0.001
DAG(40:8)	0.25(0.12,0.49)	<0.001	<0.001
PE(O-38:5)	0.17(0.07,0.40)	<0.001	<0.001
TAG(54:7/FA18:1)	0.26(0.13,0.51)	<0.001	<0.001
TAG(56:8/FA16:0)	0.26(0.13,0.51)	<0.001	<0.001
FFA(16:2)	0.27(0.14,0.52)	<0.001	<0.001
TAG(56:8/FA22:6)	0.28(0.14,0.53)	<0.001	<0.001
TAG(55:1/FA18:1)	0.32(0.18,0.56)	<0.001	<0.001
TAG(54:8/FA20:5)	0.24(0.12,0.50)	<0.001	<0.001
TAG(55:1/FA16:0)	0.20(0.09,0.46)	<0.001	<0.001
PS(38:6)	0.16(0.06,0.40)	<0.001	<0.001
TAG(54:7/FA20:5)	0.26(0.13,0.52)	<0.001	<0.001
TAG(56:7/FA18:0)	0.37(0.22,0.61)	<0.001	<0.001
TAG(55:2/FA18:2)	0.30(0.16,0.55)	<0.001	<0.001
TAG(56:9/FA20:5)	0.26(0.13,0.52)	<0.001	<0.001
TAG(56:6/FA20:5)	0.35(0.20,0.59)	<0.001	<0.001
PE(P-38:5)	0.25(0.12,0.51)	<0.001	<0.001
PE(P-38:6)	0.26(0.13,0.52)	<0.001	<0.001
TAG(56:8/FA20:5)	0.35(0.20,0.60)	<0.001	<0.001
PA(34:3)	0.18(0.07,0.44)	<0.001	<0.001
TAG(54:7/FA22:6)	0.29(0.15,0.55)	<0.001	<0.001
TAG(56:8/FA16:1)	0.36(0.21,0.61)	<0.001	<0.001
PE(34:3)	0.27(0.14,0.54)	<0.001	<0.001
TAG(56:7/FA20:5)	0.36(0.21,0.62)	<0.001	<0.001
PE(P-36:5)	0.26(0.13,0.53)	<0.001	<0.001
PA(40:5)	0.17(0.07,0.44)	<0.001	<0.001
TAG(56:8/FA18:1)	0.25(0.12,0.52)	<0.001	<0.001
DAG(34:0)	0.28(0.14,0.55)	<0.001	<0.001
TAG(54:8/FA18:2)	0.37(0.21,0.62)	<0.001	<0.001
TAG(55:7/FA22:6)	0.36(0.21,0.63)	<0.001	<0.001
TAG(53:0/FA16:0)	0.34(0.19,0.62)	<0.001	<0.001
DAG(32:0)	0.23(0.10,0.52)	<0.001	<0.001
FFA(24:1)	0.20(0.08,0.49)	<0.001	<0.001

Supplement Figure S7. The association between 36 differential lipids and risk of LC

（A） （B）

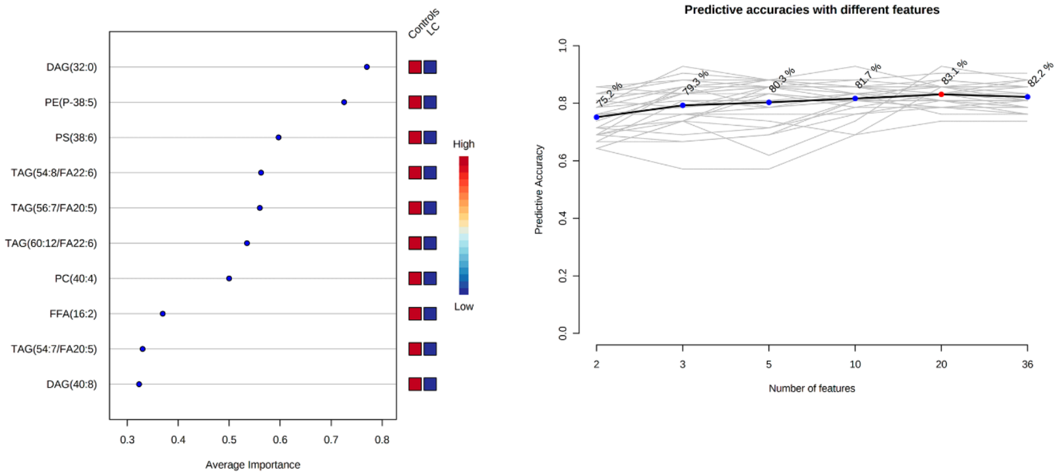

Supplement Figure S8. Selection of SVM

(A) lipidomics metabolic biomarkers of SVM classification. (B) Plot of the predictive accuracy of biomarker models (SVM)

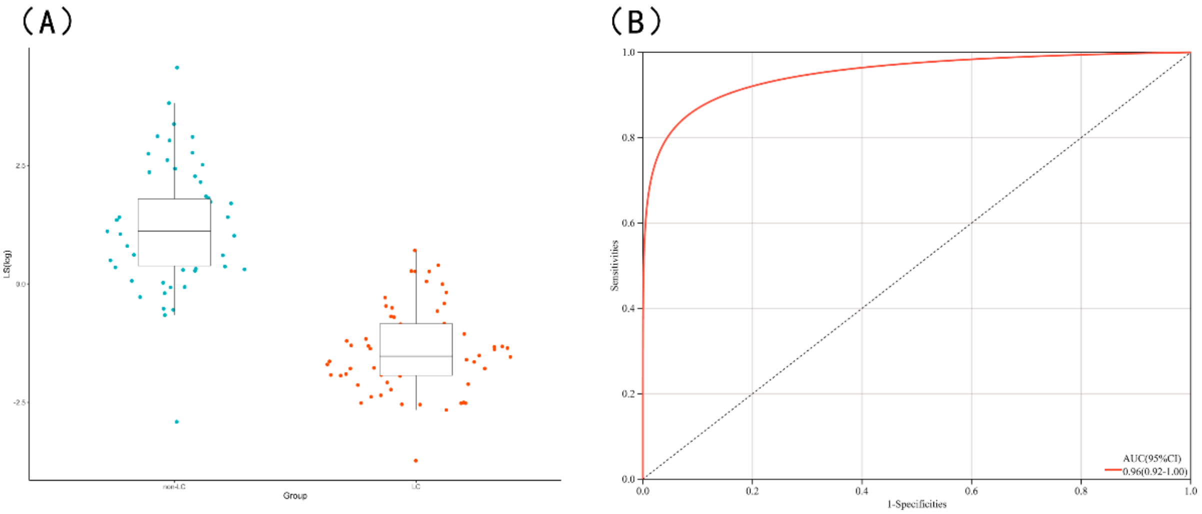

Supplement Figure S9. levels of LS in the LC and control group
(A) Box plot. (B) ROC curve analysis of LS

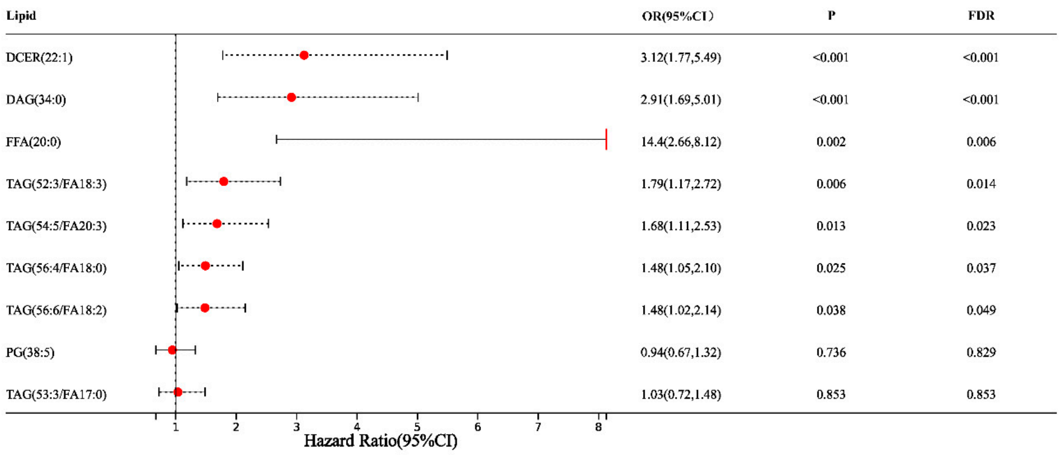

Supplement Figure S10. Nine differential lipids between early and intermediate to advanced stage groups in association with LC.

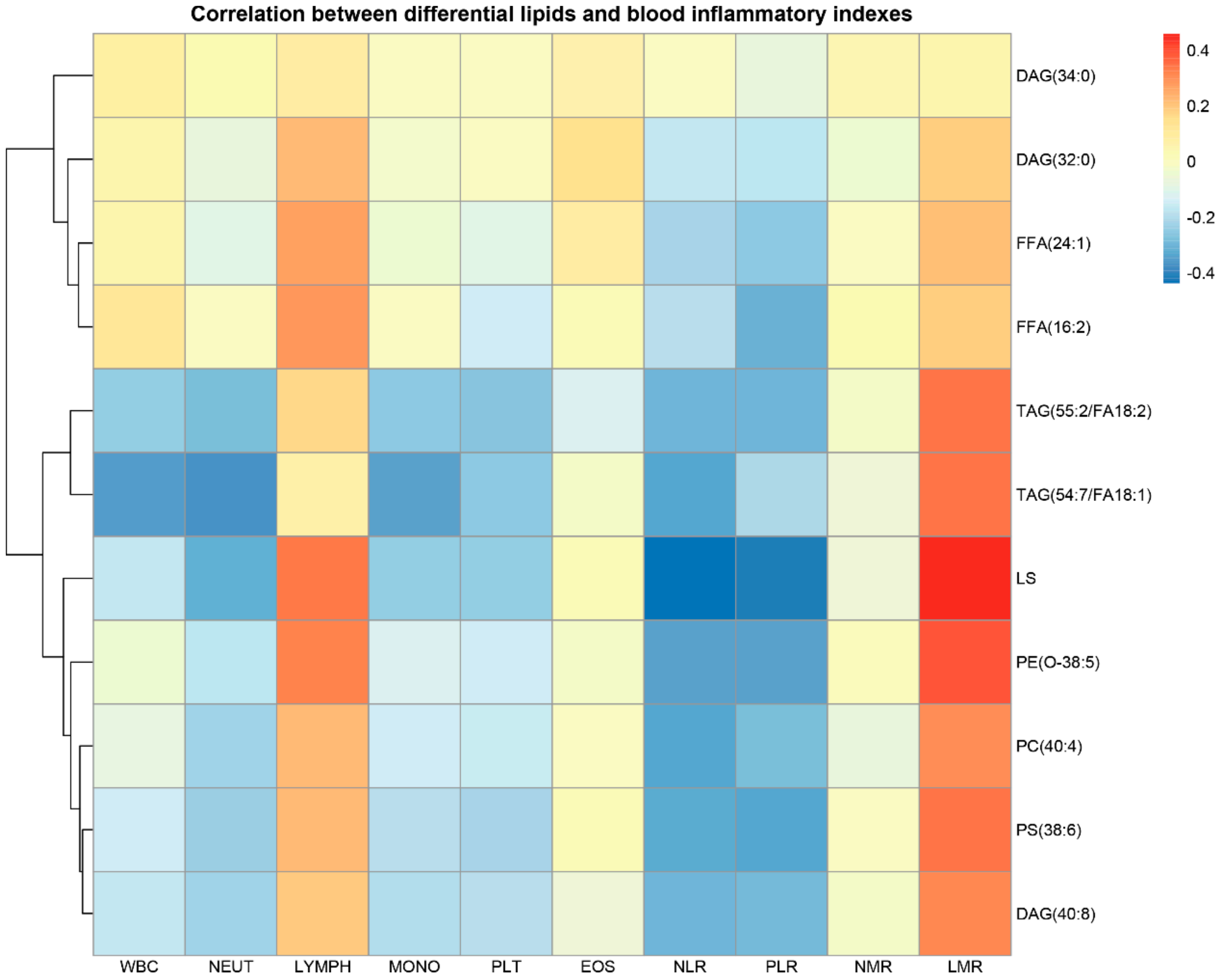

Supplement Figure S11. Heatmap of correlation between differential lipids and blood parameters
